# Screening and Management of Gestational Diabetes Mellitus after Bariatric Surgery

**DOI:** 10.3390/nu10101479

**Published:** 2018-10-11

**Authors:** Katrien Benhalima, Caro Minschart, Dries Ceulemans, Annick Bogaerts, Bart Van Der Schueren, Chantal Mathieu, Roland Devlieger

**Affiliations:** 1 Department of Endocrinology, University hospital Gasthuisberg, KU Leuven, Herestraat 49, 3000 Leuven, Belgium; caro.minschart@kuleuven.be (C.M.); bart.vanderschueren@uzleuven.be (B.V.D.S.); chantal.mathieu@uzleuven.be (C.M.); 2 Department of Obstetrics & Gynecology, University hospital Gasthuisberg, KU Leuven, Herestraat 49, 3000 Leuven, Belgium; dries.ceulemans@uzleuven.be (D.C.); roland.devlieger@uzleuven.be (R.D.); 3 Department of Development and Regeneration, KU Leuven, Herestraat 49, 3000 Leuven, Belgium; annick.bogaerts@kuleuven.be; 4 Faculty of Medicine and Health Sciences, Centre for Research and Innovation in Care (CRIC), University of Antwerp, 2000 Antwerp, Belgium; 5 Faculty of Health and Social Work, research unit Healthy Living, University Colleges Leuven-Limburg, 3001 Leuven, Belgium; 6 Department of Chronic Diseases, Metabolism and Ageing, KU Leuven, Herestraat, 49, 3000 Leuven, Belgium

**Keywords:** gestational diabetes mellitus, screening, bariatric surgery, pregnancy, obesity

## Abstract

Gestational diabetes mellitus (GDM) is a frequent medical complication during pregnancy. This is partly due to the increasing prevalence of obesity in women of childbearing age. Since bariatric surgery is currently the most successful way to achieve maintained weight loss, increasing numbers of obese women of childbearing age receive bariatric surgery. Bariatric surgery performed before pregnancy significantly reduces the risk to develop GDM but the risk is generally still higher compared to normal weight pregnant women. Women after bariatric surgery therefore still require screening for GDM. However, screening for GDM is challenging in pregnant women after bariatric surgery. The standard screening tests such as an oral glucose tolerance test are often not well tolerated and wide variations in glucose excursions make the diagnosis difficult. Capillary blood glucose measurements may currently be the most acceptable alternative for screening in pregnancy after bariatric surgery. In addition, pregnant women after bariatric surgery have an increased risk for small neonates and need careful nutritional and foetal monitoring. In this review, we address the risk to develop GDM after bariatric surgery, the challenges to screen for GDM and the management of women with GDM after bariatric surgery.

## 1. Introduction

Obesity is increasing at an alarming rate worldwide and has doubled since 1980, with a dramatic increase now also seen in low- and middle-income countries, particularly in urban settings [1]. Worldwide about 40% of adult women aged 18 years and older are overweight and 15% are obese [1]. Obesity is associated with increased risks for infertility, congenital malformations, miscarriages, GDM, preeclampsia and caesarean deliveries [1,2,3]. Severely obese women with a body mass index (BMI) ≥ 40 kg/m² or BMI ≥ 35 kg/m² with comorbidities and who obtained insufficient weight loss through lifestyle interventions, are eligible for weight-loss surgery with bariatric surgery. Since bariatric surgery is the most successful way to obtain significant and maintained weight loss, increasing numbers of obese women of childbearing age receive bariatric surgery. Women aged 18-45 years undergo now more than 50,000 inpatient bariatric surgery procedures a year in the United States, accounting for approximately half of all bariatric procedures [4]. In addition, fertility in obese women generally improves after bariatric surgery, increasing the numbers of obese women who become pregnant after surgery [4].

Gestational diabetes mellitus (GDM) was historically defined as ‘any degree of glucose intolerance with onset or first recognition during pregnancy’ [5]. Due to the increasing prevalence of type 2 diabetes mellitus (T2DM) in women of childbearing age, many international associations recommend now to screen for overt diabetes at first prenatal contact [6,7,8,9]. GDM is therefore now commonly defined as diabetes diagnosed in the second or third trimester of pregnancy provided that diabetes early in pregnancy has been excluded [7,8,9]. Women who develop GDM in late gestation, often already have a subclinical metabolic dysfunction prior to conception compared with women with normal glucose tolerance. Because of the important decrease in insulin sensitivity in normal pregnancy, the predisposing baseline insulin resistance is further exacerbated and/or in combination with beta-cell dysfunction, results in the development of GDM [10,11]. It has clearly been demonstrated that treatment of GDM between 24–28 weeks of pregnancy can reduce adverse pregnancy outcomes, especially large-for-gestational age infants (LGA) and preeclampsia [12,13]. Shortly after the delivery, the glucose values are generally restored to normal but women with GDM are at increased risk of developing T2DM later in life [14]. GDM seems also to be related to childhood obesity but mainly in case of coexisting maternal obesity [15,16]. The ‘Hyperglycaemia and Adverse Pregnancy Outcomes’ (HAPO) study showed a continuous and graded relationship between the maternal hyperglycaemia and the risk for an adverse perinatal outcome [17]. Based on the HAPO study, the ‘International Association of Diabetes and Pregnancy Study Groups’ (IADPSG) recommended a universal one-step approach with the 75g oral glucose tolerance test (OGTT) with the use of more stringent diagnostic criteria for GDM [6]. The IADPSG recommendation for GDM screening has now been endorsed by several associations including by the World Health Organization (WHO) [7,8,9,18]. Recent studies using the IADPSG screening strategy for GDM, have shown GDM rates of 25–39% in obese women [19,20]. Women often remain overweight after bariatric surgery and the risk to develop GDM is generally still higher compared to normal weight pregnant women. However, many uncertainties remain concerning screening and management of GDM in women after bariatric surgery. In this review, we address the risk to develop GDM after bariatric surgery, the challenges to screen for GDM and the management of women with GDM after bariatric surgery. As this is not a systematic review of the literature, this review is not intended to be fully comprehensive of the topic at hand. The available literature on GDM after bariatric surgery was searched in June and July 2018 through Medline and all relevant studies (without exclusion of any study type) available in English were included.

## 2. The Risk of Adverse Pregnancy Outcomes in Obese Women with GDM

GDM increases the risk of LGA, since the maternal glucose crosses the placenta and stimulates foetal insulin secretion which acts as a growth factor. LGA increases risks of shoulder dystocia and caesarean deliveries [17,21]. GDM is also associated with pregnancy-induced hypertension and pre-eclampsia. Short-term risks for the baby include neonatal hypoglycaemia, hyperbilirubinaemia and respiratory distress syndrome [17,21]. As obesity is not only associated with hyperglycaemia but also with hyperlipidaemia, lipids may provide additional substrates for foetal overgrowth. This is confirmed by recent studies showing that in GDM there is a preferential activation of lipid genes in the placenta in contrast to the activation of glucose metabolic pathways [10]. Several studies have shown that both maternal GDM and obesity are independently associated with adverse pregnancy outcomes and that the combination of obesity and GDM shows a greater risk of adverse pregnancy outcomes than either obesity or GDM alone [22,23]. Since the placenta and other tissues may already be programmed in early pregnancy, achieving optimal metabolic health before a planned pregnancy, might be the best option to obtain good pregnancy outcomes. Although several randomized controlled trials (RCT’s) investigating lifestyle interventions in overweight or obese women during pregnancy, resulted in a significant reduction in gestational weight gain, this was generally not associated with a reduction in the rate of GDM or with improvement of other pregnancy outcomes [19,20,24,25]. Women with overweight or obesity should therefore optimally lose weight before pregnancy to improve pregnancy outcomes.

## 3. Bariatric Surgery Procedures

There are three different types of bariatric surgery procedures: restrictive, malabsorptive and combined restrictive–malabsorptive procedures. Restrictive procedures, such as sleeve gastrectomy, vertical banded gastroplasty (VBG) and adjustable gastric banding (ABG), promote weight loss by decreasing the gastric volume and thereby limiting food intake. They are generally considered safer and less complicated to perform but they do have the disadvantage that high-energy foods may bypass the restriction [4,26]. Malabsorptive procedures, such as biliopancreatic diversion (BPD), cause weight loss by bypassing a significant portion of the small bowel but they induce important malabsorption. Purely malabsorptive procedures are therefore now rarely performed [4,26]. Worldwide, most procedures are currently of the combined type, with the laparoscopic Roux-en-Y gastric bypass (RYGB) [4]. RYGB is in general superior to restrictive procedures in terms of excess weight loss but is associated with more surgical complications [4]. The Swedish obese subjects (SOS) study has shown an average maximum weight loss after 1–2 years of 32% for RYGB, 25% for VBG and 20% for AGB as compared to a 1–2% change in body weight resulting from conventional lifestyle interventions [27]. A 10-year follow-up found weight loss had stabilized at 25%, 16% and 14%, respectively, for the three procedures [27].

### 3.1. Pregnancy Outcomes after Bariatric Surgery

There is now evidence that bariatric surgery performed before pregnancy is not only associated with several benefits but also with some important harms. Bariatric surgery can lead to complications during pregnancy such as internal hernias, intestinal obstructions, hyperemesis, cholelithiasis and problems with position and function of the gastric band that may require revision [28]. A high index of suspicion for gastrointestinal surgical complications is therefore needed in women after bariatric surgery who present with abdominal pain during pregnancy [29].

In addition, several nationwide register-based cohort studies have shown that bariatric surgery is associated with lower risks for foetal macrosomia, GDM and hypertensive disorders but with increased rates of small-for-gestational age (SGA) infants and preterm delivery [30,31,32]. The most recent systematic review and meta-analysis from 2018 included 20 cohort studies (with 8364 women who had bariatric surgery) and pooled odds ratios (OR) for each outcome were estimated with the use of the Dersimonian and Laird random effects. To aid interpretation, the authors also computed the pooled absolute risk difference and reported this as the number needed to benefit or the number needed to harm. This systematic review showed that bariatric surgery, with patients matched for pre-surgery BMI, resulted in a significant reduction of LGA infants [OR 0.31 (95% confidence interval (CI) 0.17–0.59)], with a number needed to benefit of 5, gestational hypertension [OR 0.38 (95% CI 0.19-0.76)], with a number needed to benefit of 11 and caesarean delivery rates [OR 0.50 (95% CI 0.38–0.67)], with a number needed to benefit of 9 [33]. This systematic review also confirmed that bariatric surgery is associated with a significant increase in SGA infants [OR 2.16 (95% CI 1.34–3.48)], with a number needed to harm of 21 and preterm deliveries [OR 1.35 (95% CI 1.02–1.79)], with a number needed to harm of 35. No differences in rates of preeclampsia, neonatal intensive care unit admissions, stillbirths, malformations and neonatal deaths were seen [33]. This suggests that bariatric surgery is not risk free and regular foetal monitoring during pregnancy is therefore warranted. Given that the risks of SGA infants and preterm deliveries were also increased when matching for pre-pregnancy weight, suggests that bariatric surgery as such is associated with adverse obstetric outcomes and not only through weight reduction [33]. 

Few studies have compared pregnancy outcomes between different type of bariatric surgery procedures and they generally showed comparable outcomes between malabsorptive and restrictive procedures [34,35,36]. However, a secondary analyses of the most recent systematic review showed that malabsorptive surgeries resulted in a significantly greater increase in SGA infants [OR 2.39 (95% CI 1.94–2.94)] and a significantly greater decrease in LGA infants [OR 0.28 (95% CI 0.22–0.36)] compared with restrictive surgeries [33]. Since the risk for SGA associated with bariatric surgery cannot only be attributed to the weight loss, this may also be related to micronutrient deficiencies during pregnancy [37]. This could therefore also explain the higher risk for SGA infants in malabsorptive procedures compared to restrictive procedures.

### 3.2. The Risk for GDM after Bariatric Surgery

Most studies have shown that bariatric surgery can significantly decrease the risk for GDM compared to the preoperative risk or compared to obese controls [28,30,32,38,39,40,41,42,43,44,45,46,47,48,49]. Table 1 gives an overview of the studies evaluating the risk for GDM after bariatric surgery since 2010. Some studies have demonstrated that bariatric surgery can decrease the risk for GDM to a rate equivalent with the general population [30,44,49] while other studies still showed a higher risk for GDM in women after bariatric surgery compared to normal weight controls [28,42,43]. The reduction in the rate of GDM also persists into a consecutive postoperative pregnancy [41]. The most recent systematic review matching to pre-bariatric surgery weight, showed that bariatric surgery resulted in a significant reduction in GDM with an OR of 0.21 (95% CI 0.11–0.37) with a number needed to benefit of 5 [33]. When women were matched for pre-pregnancy BMI, bariatric surgery was no longer associated with a lower rate of GDM [33]. This suggests that the lower risk for GDM is primarily due to weight loss rather than due to a specific mechanism inherent to the postoperative status. The interval from surgery to conception does not appear to have a significant effect on the rate of GDM as similar rates of GDM reduction were seen during and after the first year after bariatric surgery [39,50]. From current evidence, we can therefore conclude that bariatric surgery is an effective way to reduce the risk for GDM in obese women. However, since more than half of all women of childbearing age after bariatric surgery are still obese, the risk to develop GDM will in general still be higher compared to normal weight pregnant women [51]. All pregnant women with bariatric surgery should therefore undergo screening for GDM.

### 3.3. How to Screen for GDM after Bariatric Surgery?

There are no specific guidelines on screening for GDM in women with a history of bariatric surgery. The standard test for the diagnosis of GDM is the OGTT but this is generally not well tolerated in bariatric surgery patients. Evaluation of the tolerance of the OGTT in 128 bariatric surgery patients of which 30% were pregnant, showed adverse events in 65%, of which the most common were nausea (38%), dizziness (30%), weakness (26%) and diarrhoea (23%) [52]. The poor tolerance of the OGTT in patients with bariatric surgery is due to the high risk for early and late dumping after ingestion of the glucose load. Early dumping results from accelerated gastric emptying of hyperosmolar content into the duodenum or small bowel, followed by fluid shifts from the intravascular compartment into the intestinal lumen. Symptoms appear 10–60 min after food intake and can include abdominal pain, nausea, diarrhoea, fatigue, palpitations and tachycardia. Late dumping occurs 1–3 h after a meal, when a hyperinsulinaemic response to ingested carbohydrates produces postprandial reactive hypoglycaemia [4,51].

Dumping also causes wide variations in glucose excursions on the OGTT in pregnant women with bariatric surgery, making it very challenging to make a diagnosis. A large retrospective cohort study showed that pregnant women after RYGB had lower fasting glucose levels compared with lean, obese and BMI-matched controls but had more often a high glucose excursion at 1 h and 55% had a reactive hypoglycaemia at 2 h [53]. Other studies have confirmed high rates of reactive hypoglycaemia on the OGTT in 50–58% of pregnant women after bariatric surgery [54,55]. In addition, a recent study has shown that the risk for reactive hypoglycaemia was significantly higher in women with RYGB (83%) than among women with prior sleeve gastrectomy (54%) or ABG (12%) [55]. Preservation of the pyloric valve during the sleeve gastrectomy procedure might partly account for the lower risk for hypoglycaemia compared to RYGB. However, the risk for hypoglycaemia was still high in pregnant women with prior sleeve gastrectomy, suggesting that sleeve gastrectomy still causes important dumping in pregnancy [4]. The study also showed that the time from surgery to conception was significantly shorter among women with a reactive hypoglycaemia during the OGTT. Women with a reactive hypoglycaemia had also significantly higher rates of SGA infants (11.9% compared to 1.7%) [55]. The potential association found between hypoglycaemia and poor foetal growth warrants further investigation in larger studies. It also remains unclear whether maintaining a tight euglycaemic balance and avoiding hypoglycaemia will favourably impact foetal outcomes in women with bariatric surgery.

A recent study showed that by using the IADPSG criteria for GDM, 50% of all pregnant women with a history of bariatric surgery would be diagnosed with GDM but this diagnosis did not affect pregnancy outcomes [54]. These data suggest that alternative diagnostic criteria might be needed for women with bariatric surgery. Current evidence therefore suggest that an OGTT with the IADPSG criteria should not be used for screening and diagnosis of GDM in women with bariatric surgery, as this leads to inaccurate results and an increased risk for a reactive hypoglycaemia [51,54]. Moreover, this will lead to a high prevalence of GDM in women with bariatric surgery without clear evidence that treatment has a favourable impact on pregnancy outcomes.

Several professional associations recommend a two-step approach for screening for GDM, using a non-fasting 50 g glucose challenge test (GCT) to determine whether an OGTT should be performed [56]. The GCT is easier to perform and is generally better tolerated than an OGTT [57]. The GCT could therefore be used as an alternative to the OGTT in women with bariatric surgery. However, there are currently no data available on the tolerance of the GCT in women with bariatric surgery nor on potential glucose variations after the GCT in this population.

A small study has reported that continuous glucose monitoring (CGM), used in pregnant women with RYGB for 3–7 days at 24–28 weeks, showed similar glycaemic profiles as described in non-pregnant RYGB patients [58]. The authors suggest to use the time spent above 7.8 mmol/L as a surrogate for the diagnosis of impaired glucose tolerance during pregnancy after bariatric surgery since foetal exposure to such glucose excursions have been associated with foetal overgrowth [58]. Larger studies are needed to confirm these data and validate CGM thresholds for the diagnosis of GDM. In addition, CGM is expensive and not widely available [51]. No data are yet available on the potential usefulness of the Freestyle Libre Flash glucose monitoring system for the diagnosis of GDM after bariatric surgery. A case report has shown that the Flash sensor might be helpful in the management of dumping syndrome with hypoglycaemia in a pregnancy after RYGB [59].

**Figure 1:**
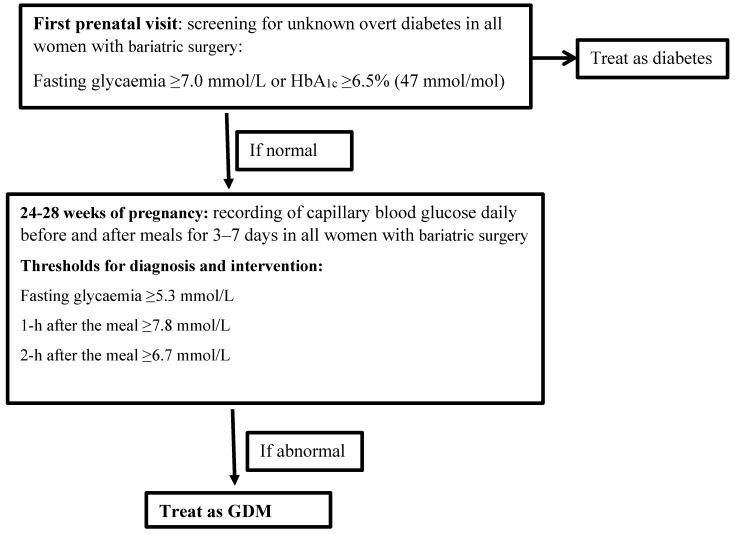
A pragmatic proposal for the evaluation of dysglycaemia in pregnant women after bariatric surgery. GDM: gestational diabetes mellitus.

Since there is currently lack of adequate tests to screen for GDM, we suggest a pragmatic approach to evaluate dysglycaemia in pregnant women with bariatric surgery (Figure 1). Since women with bariatric surgery often remain overweight, we first recommend to screen for overt diabetes at first prenatal visit by measuring the fasting glycaemia or glycated haemoglobin (HbA1c). HbA1c has the advantage that it can used in the non-fasting state but it is less sensitive than fasting glycaemia to screen for T2DM. HbA1c is also not recommended for screening for GDM as it lacks sensitivity [7]. In addition, the HAPO study showed that associations with adverse outcomes were significantly stronger with glucose measures than with Hba1c [17].

Since women with bariatric surgery generally remain at high risk for GDM, we suggest to evaluate dysglycaemia later in pregnancy in all women with bariatric surgery by recording capillary blood glucose daily before and after meals during 3–7 days at 24–28 weeks of pregnancy [51]. For the diagnostic and intervention glycaemic targets, we propose to use the same targets for the treatment of GDM as recommended by the American Diabetes Association (ADA) (fasting glycaemia < 5.3 mmol/L, 1-h after the meal <7.8 mmol/L or 2-h after the meal <6.7 mmol/L) [60]. CGM and capillary blood testing have not yet demonstrated to favourably impact on pregnancy outcomes. However, capillary blood glucose measurements may currently be the most acceptable alternative to the OGTT for screening in pregnancy after bariatric surgery [51]. More research is needed to better define dysglycaemia in a pregnancy after bariatric surgery.

### 3.4. Treatment of GDM after Bariatric Surgery

Initial treatment of GDM involves diet modification, glucose monitoring and moderate exercise. There is a paucity of evidence-based data concerning nutritional treatment for the general population with GDM. In general the recommendation is to follow the National Academy of Medicine revised guidelines from 2009 for weight gain during pregnancy, to reduce the caloric intake of obese women by approximately one-third while maintaining a minimum intake of 1600–1800 kcal per day, to limit the carbohydrate intake to 35–45% of total calories without compensatory increased intake of saturated fat, to avoid the intake of carbohydrates with a high glycaemic index and to perform daily moderate exercise for 30 min or more [60,61].

If lifestyle is insufficient to maintain the glycaemic targets after 1–2 weeks, pharmacological therapy with insulin becomes necessary. Recent studies have suggested that metformin and glibenclamide (glyburide) may be safe and acceptable alternatives for insulin for the treatment of GDM [61]. However, the need for supplemental insulin in women on metformin is high and increased risks for neonatal hypoglycaemia and macrosomia have been reported in women treated with glibenclamide [62,63]. Moreover, there is a paucity of long-term follow-up data on children exposed to oral agents in utero and recent data suggest that exposure to metformin in utero might significantly increase the risk for overweight and obesity in 4-year offspring [64]. Insulin remains therefore the first choice when lifestyle measures are insufficient to achieve good glycaemic control.

Since women with bariatric surgery, have a higher risk for intrauterine growth restriction (IUGR) and SGA neonates, questions remain as to whether the management of GDM should be adapted for women with bariatric surgery. Data are needed on the most appropriate glycaemic targets for the treatment of GDM after bariatric surgery. It is currently unclear whether the glycaemic targets which are currently commonly used and recommended by the ADA, are also adequate for women after bariatric surgery or might be too strict and further enhance the risk for SGA neonates [60]. There are currently no RCT’s that have investigated the impact of different glycaemic targets for the treatment of GDM on pregnancy outcomes in women after bariatric surgery.

### 3.5. Nutritional Monitoring and Supplementation

Bariatric surgery, in particular the malabsorptive procedures, lead to a high risk for deficiencies of several micronutrients (mainly vitamin B12, vitamin D and other fat-soluble vitamins, folate, calcium, iron and other trace elements) and macronutrients (mainly proteins and fat) [65,66]. Therefore, dietary counselling and supplementation are mandatory after bariatric surgery but compliance with follow-up remains difficult in this patient population. Due to concerns for potential nutritional deficiencies and adverse effects on the foetus during the period of rapid weight loss that typically lasts 6–18 months after surgery, it is generally recommended to avoid pregnancy following bariatric surgery for 12–24 months [4]. A large population-based retrospective cohort study showed that an operation-to-birth interval less than 2 years was associated with higher risks for prematurity, admission on the neonatal intensive care unit and SGA infants compared with longer intervals [67]. In contrast, several smaller studies found no association between a shorter time to conception and an increased risk for adverse pregnancy complications [32,36,39,68,69].

Several studies have evaluated specific evidence of micronutrient deficiencies in postoperative mothers and infants. However, these studies had small sample sizes and rarely compared them to a control population [70,71,72,73]. A higher risk for anaemia has been demonstrated in women after bariatric surgery (OR 3.4, 95% CI 1.56–7.44) and reached 15% after RYGB and 24% after BPD in a retrospective cohort of 115 subjects [70]. Low levels of vitamin B12 have also been reported in 11% to 15% of patients but the levels were not significant lower compared to a control population [71]. A prospective cohort study including 49 pregnant women with bariatric surgery showed that low circulating vitamin K1 is common and that supplementation during pregnancy can restore vitamin K1 in women with bariatric surgery, potentially protecting the foetus and new-born against intracranial haemorrhage [73]. A systematic review showed that the most common adverse neonatal outcomes related to maternal micronutrient deficiencies include visual complications (vitamin A), intracranial haemorrhage (vitamin K1), neurological and developmental impairment (vitamin B12) and neural tube defects (folate) [37]. However, the overall evidence in the systematic review was low as studies often did not provide sufficient information. The evidence on micronutrient deficiencies in pregnant and postpartum women after bariatric surgery and subsequent adverse neonatal outcomes remains therefore inconclusive. Nutritional supplementation may also not improve all pregnancy outcomes [74].

Guidelines generally recommend that patients who become pregnant following bariatric surgery should have nutritional surveillance and laboratory screening for deficiencies every trimester, including tests for iron, folate and B12, calcium and fat-soluble vitamins [65,66]. However, practice guidelines do not suggest specific supplementation during pregnancy and standard supplementation is generally recommended as for non-pregnant patients after bariatric surgery (Table 2) [65]. Based on the best available evidence and our clinical experience, we propose some suggestions for monitoring and supplementation of micronutrients in all women regardless of the type of bariatric surgery from preconception to the postpartum period (Table 3) [37].

## 4. Conclusions

Bariatric surgery is an effective way to reduce the risk for GDM in obese women. However, the risk of developing GDM is in general still higher in women after bariatric surgery compared to normal weight pregnant women. Standard screening with an OGTT is often not well tolerated and wide variations in glucose excursions make the diagnosis difficult. Capillary blood glucose measurements may currently be the most acceptable alternative for screening for GDM in pregnancy after bariatric surgery. Since women with bariatric surgery, have a higher risk for small neonates due to nutrient deficiencies, questions remain as to whether the management of GDM should be adapted for women with bariatric surgery. In particular, data are needed on the most appropriate glycaemic targets for the treatment of GDM to favourably impact pregnancy outcomes. More data are also needed on the optimal nutritional monitoring and supplementation strategy in preconception, during pregnancy and in the early postpartum period. The lack of specific guidelines concerning screening and management of GDM in women with bariatric surgery, highlights the need for more research for a better screening test and a better understanding of how to define and treat dysglycaemia in a pregnancy after bariatric surgery.

## Figures and Tables

**Table 1:** Overview of studies evaluating the risk for GDM after bariatric surgery since 2010.

Study	Year	Country	Intervention Group	Comparison Group	GDM Cases N (%)	GDM Controls N (%)
Burke et al. [38]	2010	USA	354 postoperative pregnancies	346 preoperative pregnancies	28 (8.0)	94 (27.0)
Santulli et al. [28]	2010	France	24 pregnancies after RYGB	120 normal BMI controls 120 BMI-matched controls	2 (8.3)	6 (5.0) 10 (8.3)
Lapolla et al. [49]	2010	Italy	83 pregnancies after LAGB	120 obese women 858 normal controls	5 (6.0)	60 (50.0) NR
Sheiner et al. [39]	2011	Israel	104 pregnancies < first postoperative year	385 pregnancies > first postoperative year	11 (10.5)	28 (7.3)
Aricha-Tamir et al. [40]	2012	Israel	144 postoperative pregnancies	144 preoperative pregnancies (same women)	8 (5.7)	28 (19.3)
Josefsson et al. [42]	2013	Sweden	310 firstborns after surgery	270,805 Swedish firstborns	17 (5.9)	3.514 (1.4)
Kjaer et al. [32]	2013	Denmark	339 postoperative pregnancies	1277 matched controls	30 (8.9)	91 (7.1)
Amsalem et al. [41]	2014	Israel	109 first pregnancies postoperative 109 s pregnancies postoperative	109 preoperative pregnancies	6 (5.6) 7 (6.6)	21 (19.0)
Berlac et al. [43]	2014	Denmark	415 singletons after RYGB	827 adipose controls 829 normal weight controls	38 (9.2)	67 (8.1) 11 (1.3)
Shai D et al. [46]	2014	Israel	326 postoperative pregnancies	1612 obese controls	33 (10.1)	237 (14.7)
Johansson et al. [30]	2015	Sweden	596 postoperative births	2356 matched control births	11 (1.9)	157 (6.8)
Adams et al. [44]	2015	USA	295 women with births before and after RYGB	295 control births	10 (3.4)	26 (8.8)
Abenhaim A et al. [45]	2016	Canada	9587 postoperative pregnancies	8,244,661 controls 221,580 morbid obese controls	1011 (10.5)	224,758 (2.7) 18,936 (8.5)
Parker MH et al. [47]	2016	USA	1585 postoperative pregnancies	185,120 obese controls	119 (7.3)	8145 (4.4)
Chevrot A et al. [48]	2016	France	139 postoperative pregnancies (58 RYGB, 81 LAGB and 9 sleeve gastrectomy)	139 obese controls matched for pre-surgery BMI	17 (12)	32 (23)

GDM: gestational diabetes mellitus; RYGB: laparoscopic Roux-en-Y gastric bypass; LAGB: laparoscopic adjustable gastric banding; BMI: body mass index (kg/m²); NR: not reported.

**Table 2:** Recommended nutritional supplementation after bariatric surgery.

Nutrients	Recommended Supplementation
Multivitamin tables (with iron, folic acid and thiamine)	2 tables/day
Calcium	Calcium citrate 1200–1500 mg/day
Vitamin D 3000 IU/day	3000 IU/day titrated to 25-hydroxyvitamin D level
Vitamin B12 as needed	As needed for normal range
Iron supplement	45–60 mg/day
Protein intake	Minimal 60 g/day and up to 1.5 g/kg ideal body weight per day
Vitamin A	5000–10 000 IU/day

**Table 3:** Proposal of micronutrient monitoring and supplementation from preconception to postpartum in women after bariatric surgery.

	Timing of Screening	Monitoring of Micronutrients	Supplementation
Preconception	Every 6 months	Vitamin A (preferably as Beta carotene), D, B12, folate, K1 and iron	Multivitamin tablet with vitamin B12 and folate with additional supplements as needed
During pregnancy	Every trimester and additional screening if low levels despite supplement	Vitamin A (preferably as Beta carotene), D, B12, folate, K1 and iron	Multivitamin tablet with vitamin B12 and folate with additional supplements as needed
Postpartum	At 6–12 weeks in all women additional screening at 3–6 months if low levels despite supplement yearly follow-up if no deficiencies at 6–12 weeks	Vitamin A (preferably as Beta carotene), D, B12, folate, K1 and iron	Multivitamin tablet with vitamin B12 and folate with additional supplements as needed
